# Culture hidden in plain sight: a framework-based secondary qualitative analysis of clinicians’ experiences with the cultural formulation interview

**DOI:** 10.3389/fpsyt.2026.1895098

**Published:** 2026-09-03

**Authors:** Ankita Deshmukh, Sheetal Lakhani, Vasudeo Paralikar

**Affiliations:** 1 Butterfly Psychotherapy Clinic, Pune, India; 2 Psychology, Vidyashilp University, Bengaluru, India; 3 Psychiatry, King Edward Memorial (KEM) Hospital & King Edward Memorial Hospital Research Centre (KEMHRC), Pune, India

**Keywords:** clinical implementation, clinician perspectives, cultural competence, cultural formulation interview, cultural psychiatry, person-centered psychiatry, secondary qualitative analysis, sociocultural assessment

## Abstract

When patients describe their family obligations, financial pressures, and relational worlds during a Cultural Formulation Interview (CFI), clinicians often listen carefully - and then wonder what any of it has to do with culture. This, the present review found, is at the heart of the persistent CFI implementation gap. Despite broadly favorable evaluations of the DSM-5 CFI, clinician challenges in routine implementation have persisted across settings, versions of the instrument, and levels of institutional investment. Most explanations have attributed this gap to logistical barriers such as time, workload, and instrument rigidity. To understand not just what clinicians report but also how and why they experience the CFI as they do, we conducted a framework-based secondary qualitative content analysis- synthesizing clinician narratives and perspectives from six field-trial studies (FTS) and four post-field-trial studies (PFTS). We mapped perspectives from 128 clinicians across nine countries onto the evaluative domains of feasibility, acceptability, and clinical utility, comparing findings across the two study groups to identify what has persisted, what has shifted, and what remains unaddressed. Ten themes emerged across the FAU domains. Seven recurred across both FTS and PFTS - suggesting that core implementation challenges are structural rather than incidental, and have not been substantially resolved by successive revisions to the instrument or its training. Two themes emerged distinctly in the PFTS - systemic and organizational barriers, and clinician factors shaping utility - reflecting a deepening in the field’s understanding of what drives implementation success beyond logistics. The central finding cuts across all domains: clinicians consistently elicited sociocultural information, however many of them failed to recognize it as cultural. When they did recognize it, clinicians seemed unsure how to use such information in formulation and treatment planning. In this sense, culture appeared hidden in plain sight: present within clinical encounters, offered by patients, but not consistently identified or acted upon as culturally meaningful, perhaps missed by clinicians whose training had organized their attention elsewhere. While the CFI’s contribution to rapport and person-centered care is well documented, its potential role in treatment planning has received comparatively little attention. The implementation gap, this review suggests, is less a problem of feasibility than of conceptual clarity: clinicians need a more nuanced understanding of what culture is, where it appears, and how to utilize it best. Recommendations synthesized across the literature - organized from minimal instrument refinements to transformative reconceptualization of psychiatric assessment - point consistently toward training that cultivates this understanding rather than merely teaching a procedure. The authors additionally propose a broader conceptualization of culture for use in CFI training contexts, and call for future training models that empower clinicians to recognize, interpret, and utilize sociocultural information as part of routine clinical reasoning.

## 1 Background

The Cultural Formulation Interview (CFI) of the DSM-5 has continued to garner scholarly and clinical interest over the decade since the International Field Trials (1). Developed from the Outline for Cultural Formulation, the CFI is a person-centered, semi-structured interview designed to systematically elicit information about the cultural and contextual dimensions of a patient’s clinical presentation across four domains: the definition of the problem; perceived causes, context, and sources of support; self-coping and past help-seeking; and current help-seeking and expectations of care. The CFI represents a significant step toward integrating culturally responsive practices into routine psychiatric assessments and has brought discussions of cultural competence into mainstream discourse, facilitating the co-construction of illness narratives with patients.

The landmark multi-site field trial by Lewis-Fernandez et al. established a framework using three evaluative constructs- feasibility, acceptability, and clinical utility (FAU)- to assess the CFI’s value in clinical practice (1). Feasibility referred to whether the interview could be administered within routine settings; acceptability referred to whether patients and clinicians found the interview appropriate and agreeable; and clinical utility referred to whether the CFI generated information useful for diagnosis, treatment planning, and therapeutic engagement (1). These were assessed using debriefing tools that included both quantitative Likert-scale ratings and qualitative narratives. Overall, the field trial found the CFI to be feasible, acceptable, and clinically useful across diverse settings; however, clinician ratings were lower than those of patients. Qualitative findings also noted clinician concerns regarding administration time and the difficulty of certain questions, despite their appreciation for the tool’s rapport-building potential (1).

Subsequent studies across varied healthcare systems and populations have largely supported these findings, further documenting the value of the CFI in enhancing rapport and eliciting patient perspectives (2–12). A recent systematic review by Kayrouz et al. (2024) synthesized evaluation ratings of the CFI across patients, clinicians, and relatives in five studies involving 581 participants (13). While the review confirmed broad satisfaction with the CFI, it also highlighted that feasibility concerns persist as a consistent challenge for clinicians. Although this work made an important contribution in synthesizing quantitative estimates of feasibility, acceptability, and clinical utility (FAU), the authors noted the need for methodologically richer approaches to understand continuing implementation challenges. Indeed, while the value of cultural assessments is broadly acknowledged, their clinical uptake remains variable and uneven across settings (3, 4, 10).

Beyond quantitative data, few studies have gathered qualitative insights into stakeholder experiences across the domains of feasibility, acceptability, and utility. Clinician experiences provide a crucial ground-level account of implementation; as the primary agents of CFI use, their narratives are essential to understanding why an interview widely praised for its acceptability remains underutilized. Existing qualitative work highlights barriers such as conceptual confusion regarding cultural information, limited buy-in from practitioners, and uncertainty in translating CFI findings into clinical practice (3, 5, 14). However, these findings remain scattered across studies with disparate designs, settings, and versions of the CFI, making it difficult to discern consistent patterns. To our knowledge, no attempt has been made to synthesize this rich qualitative data to develop a holistic understanding of the implementation experience.

A recent state-of-the-science review by Aggarwal (2024) examined a closely related but distinct question: whether and how clinicians have used information from the CFI to produce cultural case formulations, specifically in the clinical tasks of diagnostic reasoning and treatment planning (15). Using inductive content analysis across twelve studies, the author found that ten reported the CFI helpful for at least one formulation task, and identified clinicians’ preexisting conceptions of culture as an underexplored influence on clinical decision-making- a direction proposed for future research. The present review addresses this identified gap through a framework-based secondary qualitative content analysis of clinician narratives, organized within the FAU evaluative framework established by the CFI field trial. Rather than examining what clinicians have done with CFI data in cases where formulation occurred, we examine the qualitative clinician experience across the evaluative domains of feasibility, acceptability, and utility, and what those experiences reveal about the conceptual and pedagogical factors that influence whether cultural information is recognized and translated into clinical reasoning.

The body of CFI literature can be organized into field-trial studies (FTS) and post-field-trial studies (PFTS). The original FTS followed a shared design across eleven sites, evaluating a single 14-item version of the CFI against a common goal: examining the implementation experience of clinicians and patients. Post-field-trial studies, by contrast, vary considerably in methods, aims, clinical context, and the version of the CFI studied. This heterogeneity complicates conventional pooled analysis but makes a qualitative synthesis of clinician narratives particularly valuable- allowing recurring patterns to be traced across a body of work that shares a common evaluative framework (FAU), even where study design and instrumentation diverge.

The present review therefore aims to analyze the thematic content of published clinicians’ experiences with the CFI across both FTS and PFTS, organized within the evaluative framework of feasibility, acceptability, and clinical utility, and to examine the broader cultural and contextual factors that shape those experiences. By comparing clinician narratives across these two bodies of literature- which differ in purpose, clinical context, training conditions, and geographical setting- we aim to illuminate what is context-dependent and what is structurally persistent in clinician experience of the CFI.

## 2 Materials and methods

### 2.1 Study design

This review employed a framework-based secondary qualitative content analysis of published clinician narratives and qualitative findings from empirical studies examining the implementation and clinical use of the Cultural Formulation Interview (CFI) as reported in field-trial studies (FTS) and selected post-field-trial studies (PFTS). The framework used to organize the data and report the thematic content was based on the feasibility, acceptability, and clinical utility (FAU) evaluative parameters established by Lewis-Fernández et al. (1) in the international field trial of the CFI. This framework was selected because it represents the established evaluative standard in CFI research and provided a meaningful structure through which clinician experiences could be systematically compared across the two corpora of studies.

### 2.2 Data sources and study selection

#### 2.2.1 Search strategy and databases

A targeted literature search was conducted across two electronic databases: PubMed and Google Scholar. In PubMed, the following search strategy was used: *(“Cultural Formulation Interview”) AND (clinician OR psychiatrist OR provider* OR practitioner* OR psychologist*) AND (qualitative OR interview* OR perspective* OR experience* OR implementation)*. This search yielded 72 records. Google Scholar was searched using multiple predefined focused title searches because it does not reliably support complex Boolean combinations. The following title searches were performed independently: *allintitle:”Cultural Formulation Interview” clinician*, *allintitle:”Cultural Formulation Interview” qualitative*, *allintitle:”Cultural Formulation Interview” perspective*, *allintitle:”Cultural Formulation Interview” experience*, and *allintitle:”Cultural Formulation Interview” implementation*. This search yielded 20 additional records. Abstracts and full texts were screened using the following criteria, retaining ten final papers that are included in the review.

#### 2.2.2 Inclusion and exclusion criteria

Studies were eligible if they were (i) empirical investigations conducted in adult psychiatric settings and that (ii) reported qualitative clinician narratives, quotations, or author-interpreted qualitative findings regarding the implementation or clinical use of the CFI. Studies were excluded if they were theoretical articles, review papers, systematic reviews, meta-analyses, book chapters, quantitative-only studies, studies focusing exclusively on patients’ or families’ perspectives without clinician narratives, studies conducted in child and adolescent or neurological settings, forensic or asylum-seeker populations, or studies evaluating cultural assessment tools other than the DSM-5 CFI. Studies focusing exclusively on a specific diagnosis (e.g., grief, Post Traumatic Stress Disorder) were excluded.

A total of 10 papers were included in the review. The objective was not exhaustive retrieval of all CFI-related publications but the identification of empirical studies that reported qualitative clinician data relevant to experiences with implementing or using the CFI. It is acknowledged that the depth and granularity of qualitative clinician data varied considerably across included studies, ranging from extended verbatim quotations to brief author-summarized findings; this variation is addressed in the data analysis section and noted as a limitation. The FTS results with clinician narratives were published in six papers, a pooled data paper and five studies across three sites as indicated in **Table 1** (1, 11, 14, 16–18). A total of four PFTS studies from four different countries were selected (2, 3, 5, 8), focusing on or presenting findings on the qualitative exploration of clinician perspectives as well as implementation facilitators and barriers. The PFTS papers examined feasibility, acceptability, and utility (Mexico, Sweden), clinician experiences with CFI (Sweden) and the cultural identity sections of the CFI (Denmark), and perceptions and barriers related to implementation (the Netherlands). Details of the selected studies are presented in **Table 1**.

**Table 1:** Summary of clinician characteristics and Cultural Formulation Interview (CFI) administration across included studies.

Study reference	Study site	Number of clinicians	Number of CFI administrations	Composition of clinicians	Clinician training in the CFI
Field trial papers					
Lewis-Fernández et al., 2017 (1)	11 sites, 6 countries	75 (across all sites)	318 patients (across all sites; some secondary sources report 321)	37 psychiatrists and trainee psychiatrists, 21 psychologists, 11 social workers, 6 ‘other mental health clinicians’	Standardized field trial training protocol, shared across the field trial sites as part of the coordinated multi-site DSM-5 CFI field trial (1).
Aggarwal et al., 2013; Aggarwal et al., 2015 (14, 11)	USA: 2 psychiatric sites and 1 community service site	7	32	3 psychiatrists, 1 psychologist, 2 social workers, 1 other	
Paralikar et al., 2020; Paralikar et al., 2015 (16, 17)	Pune, India	10	36	7 psychiatrists, 1 trainee psychiatrist, 2 clinical psychologists	
Rohlof et al., 2017 (18)	Netherlands	11	30	2 psychiatrists, 6 psychologists, 3 social psychiatric nurses	
Post field trial papers					
Ramírez Stege & Yarris, 2017 (2)	Mexico	11	19	1 psychiatrist, 10 psychiatry residents	Brief introductory explanation (translated from DSM-5 version).
Wallin et al., 2020 (8)	Sweden	15	114	Psychologists, psychiatric nurses, counsellors and psychotherapists (exact numbers by profession not reported)	Two half-day courses with lectures, role-play, and case discussion.
Lindberg et al., 2022 (5)	Denmark	17	20	8 resident doctors, 4 psychiatrists, plus psychologists, nurses, social workers, and other multidisciplinary mental health professionals	1-hour training on multicultural encounters, CFI rationale, and 16-question overview.
Silvius et al., 2024 (3)	Netherlands	8	Not available	1 psychiatrist, 2 senior psychologists, 5 junior psychologists	Two training tracks: 60-minute online and supervisor-level workshop.

Across all included papers, perspectives from a total of 128 clinicians were synthesized, including 75 clinicians from the field-trial studies and 53 clinicians from the selected post-field-trial studies. Considerable variation was observed in implementation preparation across studies. Whereas the field-trial studies involved standardized CFI training, post-field-trial implementation ranged from brief introductory orientation (Mexico) to structured workshops (Sweden, Denmark) and organization-wide implementation strategies including supervisor training and electronic health record integration (Netherlands). These training details are noted in **Table 1** and are considered in the interpretation of findings.

### 2.3 Data analysis

The present analysis constitutes a secondary qualitative content analysis - defined as the re-analysis or interpretive use of qualitative data originally collected and reported within primary studies, applied here to address a research question distinct from those of the original publications (19). This approach is appropriate when primary data are available in published form and when the aim is to generate new conceptual understanding by examining patterns across studies rather than within a single dataset.

Relevant clinician narratives and paraphrased findings formed the unit of analysis. These were extracted through repeated immersion in the results and discussion sections of the included studies. Where available, direct clinician quotations were prioritized; where only author-summarized findings were reported, these were extracted and treated as secondary-level qualitative data. Efforts were made to distinguish clinicians’ expressed experiences from authors’ interpretive commentary, though the latter informed the critical sections presented in the discussion (as noted in 2.4).

Following extraction, data segments were mapped onto the evaluative domains of feasibility, acceptability, and clinical utility (FAU). Definitions adapted from Lewis-Fernández et al. (1) were further elaborated to guide the thematic mapping process:

• Feasibility: the perceived ease of administering and integrating the CFI into routine clinical systems and workflows.
• Acceptability: clinicians’ perceived likability of, interest in, agreement with, and satisfaction while using the CFI in routine clinical practice.
• Clinical Utility: the perceived usefulness of the CFI for assessment, diagnosis, treatment planning, therapeutic alliance, and contextual understanding.

In some instances, data segments reflected overlapping thematic content and were therefore mapped onto more than one domain to preserve the multidimensional nature of clinicians’ experiences. This process was conducted separately for the FTS and PFTS datasets. Data extraction, organization, and coding were carried out manually using Microsoft Excel. Themes were subsequently compared across the FTS and PFTS corpora to identify patterns of consistency, divergence, and contextual variation, with attention to differences in study purpose, clinical setting, training preparation, and geographical context rather than time alone.

Following domain mapping, the authors repeatedly re-immersed themselves in the grouped data segments to identify and refine emerging themes within each domain. Discrepancies in domain allocation were resolved through discussion until consensus was reached. Final themes were subsequently refined through agreement among the research team (**Table 2**).

**Table 2:** Themes of qualitative synthesis of clinician perspectives across field trial and post-field trial studies.

Themes	FTS	PFTS
Feasibility		
Time Constraints and Workload Burden as Persistent Implementation Barriers	✓	✓
Perceived Rigidity of the CFI Prompted Flexible, Need-based Administration	✓	✓
Systemic and Organizational Implementation Barriers	✗	✓
Patient-Related Factors in the Perceived Feasibility of the CFI	✓	✗
Acceptability		
Relational Benefits of the CFI: A Context-Dependent Driver of Acceptability	✓	✓
Interactional Discomfort with Cultural-Identity and Clinician-Patient Relationship Questions	✓	✓
Clinical utility		
CFI Deepened Contextual, Person-Centered Understanding of the Client	✓	✓
The Contribution of the CFI to Diagnosis and Treatment Planning Remained Inconsistent	✓	✓
Clinical Utility Depended on Clinician’s Conceptualization of Culture	✓	✓
Clinician Factors Shaped the CFI’s Perceived Utility	✗	✓

### 2.4 Synthesis of recommendations and interpretive analysis

We additionally incorporated two analytic activities that inform the discussion section.

First, recommendations proposed by authors across the included literature regarding CFI refinement, training, and implementation were extracted alongside clinician narratives and synthesized inductively. These were not treated as primary findings but were organized according to the degree of change they implied- ranging from minimal refinements to the existing CFI instrument, through moderate adaptations to training and workflow, to transformative or paradigm-level re-conceptualizations of psychiatric assessment. This synthesis is presented in **Table 3** and discussed in relation to the primary findings.

**Table 3:** Recommendations for CFI implementation organized by level of change required.

Minimal refinements Adjustments to CFI content and wording; preserving CFI structure	Moderate adaptations Changes to training, workflow, and implementation; CFI model intact	Major recommendations Paradigm-level reconceptualization of CFI or psychiatric assessment
**Cultural identity question (Q8-10)** Reframe cultural identity questions to reduce abstraction (5; 16; Strand & Bäärnhielm, 2021; 7; 20) **Interview introduction and framing** Modify the introduction to prepare patients for contextual questions differing from routine psychiatric interviews & incorporate collaborative framing. (5; Strand & Bäärnhielm, 2021; 7) **Abbreviated version** Develop short-forms of CFI identifying high-priority questions for time-limited & emergency settings. (9; 17) **Broader cultural prompts** Add prompts addressing migration, religion & spirituality, gender roles, family, employment, class, caste, and structural inequality. (5; 2) **Timing of administration** Modify the timing of CFI administration according to clinical context, including use during intake or later phases of assessment (14; 2) **Language and contextual adaptation** Translate and adapt the CFI for different linguistic, developmental, & clinical populations; use the CFI Informant Version to include family members. (21; 13; 17)	**Standardized training format** Combine didactic teaching with interviewing-skills training, behavioral simulations, supervised practice, & case discussions (22; 13; 5; 2; 23) **Flexibility in administration** Encourage flexible phrasing, sequencing of questions to preserve conversational flow & contextual relevance (14; 13; Strand & Bäärnhielm, 2021) **Reflexivity & cultural humility** Address clinicians’ own cultural positions & risk of essentializing culture. (5; 2) **Ongoing supervision** Provide mentorship and feedback after the first case; make training count toward continuing education. (9; 5) **Fidelity and competency assessment** Use fidelity instruments and process measures to evaluate cultural interviewing competencies beyond self-report. (6; 13; 5) **Translating findings into care** Use implementation and formulation aids (e.g., CFI-EA, McGill CCS) to support integration of sociocultural findings into formulation and treatment planning. (6, 15; 9; 13; 5; 20) **Timing and institutional support** Integrate CFI into documentation systems, residency training & institutional protocols with administrative support. (14; 5; 2; 3) **Research priorities** Evaluate the impact of the CFI on alliance, diagnosis & patient outcomes across diverse settings. (13; 12) **Integrating the CFI with the OCF** Align the CFI more comprehensively with broader OCF domains and contextual factors. (20)	**Culture as universally relevant** Dismantle the “only for minorities” framing; reframe CFI as relevant to all patients regardless of background. (13; 5; 3; 7) **Assessment as a therapeutic encounter** Apply therapeutic assessment principles to support collaborative reflection and meaningful feedback on sociocultural findings. (Strand & Bäärnhielm, 2021) **CFI as pedagogy, not procedure** Embed CFI training within a broader cultural competency curriculum; treat it as a vehicle for clinical reasoning, not a checklist. (Aggarwal et al., 2020; 3) **Decentring the CFI** Conceptualize cultural formulation as a broader clinical reasoning framework integrated throughout psychiatric assessment rather than as a standalone instrument. (23; 3) **Symptom-to-context shift** Shift psychiatric assessment from predominantly symptom-focused toward more person-centered approaches. (3) **Structures of society and culture in OCF framework** Reconceptualize the OCF to include societal & cultural structural factors; providing SCFI, the tool to facilitate the assessment. (20)

CFI, Cultural Formulation Interview; CFI-EA, Cultural Formulation Interview Engagement Aid; CCS, Cultural Consultation Service; OCF, Outline for Cultural Formulation.

Second, an interpretive and critical analytic approach was also applied to examine broader conceptual tensions emerging across the dataset. This included recurring assumptions regarding what constitutes cultural information in clinical encounters, the influence of clinicians’ own cultural and professional positioning on their experience of the CFI, and the relationship between feasibility concerns and deeper uncertainties about clinical utility. These interpretive observations were developed iteratively during the analytic process and are presented in the discussion section, where they inform a broader account of the persistent implementation challenges identified across studies and their implications for training and future practice.

## 3 Results

Qualitative findings from field-trial studies (FTS) and post-field-trial studies (PFTS) are synthesized within a feasibility, acceptability, and utility (FAU) framework. Because clinicians’ experiences with the CFI were interconnected rather than confined to a single domain, several themes recurred across more than one FAU category; these cross-cutting themes are reported separately following the domain-specific findings.

A note on how results are reported: because this is a secondary analysis synthesizing findings across multiple studies, results are attributed by country or study context — for example, “clinicians from Denmark” or “clinicians in the FTS.” This convention reflects the study from which the finding is drawn and should not be read as implying that all clinicians within that context held the same view. Findings represent patterns and perspectives reported within each study rather than uniform national or site-level positions. The FTS or PFTS designation is included throughout to support the comparative reading of themes across the two bodies of literature, and **Table 2** is intended to be read alongside the results to aid this synthesis.

### 3.1 Feasibility

#### 3.1.1 Time constraints and workload burden as persistent implementation barriers

Clinicians, across both field-trials (FTS) and post-field trial studies (PFTS), expressed concerns regarding the time required for administration and documentation within the routine workload burden and recognized this as a barrier to the feasibility of CFI implementation. The most commonly reported concern was regarding the CFI administration time conflicting with the routine consultation length. Clinicians from the USA and India (FTS), and Mexico and Sweden (PFTS) described time pressure in a fast-paced clinical setting as limiting the feasible use of the CFI. Elaborating on this facet, clinicians from the USA were also concerned whether this time investment yielded a significant contribution, as they did not see the information as diagnostically relevant. This reflects a concern that crosses into the utility domain, discussed further in section 3.3.

The large number of questions was overwhelming for clinicians from the Netherlands (PFTS). This is further complicated by additional documentation requirements for CFI implementation alongside routine clinical records, as reported by Swedish (PFTS) clinicians. Finally, clinicians from Mexico (PFTS) emphasized that sustainable implementation of the CFI over time would require long-term institutional commitment and investment, as it is not a single-encounter problem. Indian clinicians (FTS) raised a related but distinct concern about whether adequate rapport needed to be established before the CFI could be feasibly administered to elicit sensitive information.

#### 3.1.2 Perceived rigidity of the CFI prompted flexible, need-based administration

Clinicians perceived the structure of the CFI to be rigid and as constraining its feasibility, even though it is a semi-structured interview. They also proposed recommendations to help adapt. This concern was observed in the USA (FTS) and Mexico, Sweden, and Denmark (PFTS).

Clinicians in the FTS expressed the CFI’s fixed question sequence as constraining the natural flow of conversation and limiting their ability to follow the patient’s narrative. They also noted some questions to be redundant, as earlier questions had already elicited the same information. Most clinicians from the USA proposed that clinicians should be able to select relevant questions from the CFI during clinical interviews as per their discretion, or administer the CFI after their routine interview.

Clinicians in the PFTS did not directly describe these issues; it was noted that clinicians made some rephrasing questions and used additional probes to enable smoother CFI administration (Mexico and Sweden). Clinicians with multicultural clinical experience indicated a preference for unstructured interviews with patient-led flow, and indicated structured interviews may be useful for less experienced people (Mexico and Denmark).

#### 3.1.3 Systemic and organizational implementation barriers

This theme was noted in the PFTS (Mexico, Netherlands), where clinicians recognized that implementation of the CFI required both individual and organizational level efforts.

The existing healthcare system involves multidisciplinary teams, follows protocols, and has ways of communication to facilitate overall care of the patient. Use of CFI was described as competing with these institutional priorities. For example, in some setups, juniors conduct the intake assessment, which requires training. In a multidisciplinary team, conveying culturally important information becomes difficult, thus risking the loss of information. Clinicians, especially from Mexico (PFTS), indicated the need for long-term effort to bring out systemic level changes for training and implementation of the CFI in a sustainable way. On the other hand, the PFTS Netherlands study, interestingly, took a systemic approach, integrating the CFI directly into the electronic patient record for all routine intakes starting in 2017 at the Parnissia Psychiatric Institute. However, there was only a tentative implementation policy from leadership (3). Several administrators did not support the CFI rollout, believing the clinical choice should be left entirely up to individual clinicians. The training on CFI was not made mandatory. Consequently, a large proportion of clinicians did not know about the CFI and those who used it found the 16 core questions overwhelming, reducing the CFI to a restrictive, bureaucratic checklist that actively damaged clinical rapport rather than fostering collaborative engagement.

#### 3.1.4 Patient-related factors in the perceived feasibility of the CFI

Patient characteristics were discussed as potential constraints on CFI administration only in the USA (FTS). The applicability of the CFI was perceived to be limited among patients with psychosis, cognitive impairments, or low educational levels. Clinicians reported difficulty administering culturally oriented and abstract questions to these patients, who struggled to engage with reflective or context-based inquiries. These clinicians expressed concern that the interview required a degree of insight, abstract reasoning, and verbal elaboration, and that the CFI may be less feasible with individuals with severe clinical presentations. Interestingly, this finding seemed to be the least supported across clinician narratives from other studies.

### 3.2 Acceptability

#### 3.2.1 Relational benefits of the CFI: a context-dependent driver of acceptability

The potential of the CFI to enhance rapport with the patient and empathy in the clinician was seen to be a positive acceptability driver across the FTS and PFTS. For instance, clinicians acknowledged that patients felt heard and led to relational gains, which the Mexican clinicians referred to as “confianza”. These relational gains were regarded as one of the principal contributors to the CFI’s acceptability.

However, rapport alone did not seem to consistently translate into greater clinician acceptability. An unusual pattern was also observed, wherein experience with the tool didn’t improve acceptability ratings of the clinicians in the USA and the Netherlands (FTS). Additionally, clinicians reported discomfort with certain questions, such as those on cultural identity and the clinician-patient relationship, which interrupted the flow. Danish clinicians (PFTS) considered the interview acceptable only when such questions could be phrased flexibly and integrated naturally into the consultation without making the patients feel ‘othered’, whereas Swedish clinicians consistently regarded the CFI as strengthening rapport and collaborative engagement. These findings suggest that the relational benefits of the CFI are not inherent to the interview itself but depend on clinicians’ ability to administer it in a manner that preserves conversational flow and patient comfort.

#### 3.2.2 Interactional discomfort with cultural-identity and clinician-patient relationship questions

Difficulty in assessing the cultural identity question was observed consistently across the FTS and PFTS, as clinicians noted that patients often struggled to understand the meaning of the question, leading clinicians to explain it in their own words. Consequently, this limited the information elicited. In instances where in-depth information could be elicited, clinicians appreciated its value.

Despite this shared experience, clinicians differed in *why* they perceived these questions as problematic and *how* they understood the purpose and implications of these questions. Two contrasting points of view can be observed from Denmark and Sweden (PFTS) regarding the importance of assessing “cultural” questions. Danish clinicians were, to begin with, reluctant and embarrassed to ask these questions, as in their context culture can function as a proxy for race, thus risking “othering” from the outset if such questions are posed without indication in the patient’s history. They anticipated the focus shifting toward differences rather than common ground. They attributed patients’ vague answers and frequent requests for clarification to the distance created by this perception of the questions. In contrast, Swedish clinicians appreciated that these questions could be asked irrespective of immigration status, meaning the CFI was not selective in its application and reduced the risk of othering. They saw the potential of these questions to enable patients to feel heard and valued.

Challenges and discomfort with CFI questions also extended beyond cultural identity. Clinicians from the FTS expressed apprehension about the question on the patient-clinician relationship. They elaborated that patients might not be comfortable disclosing this information, as they might perceive it as socially inappropriate or may desire to be socially desirable, and therefore the authenticity of the information shared could be questioned. These findings suggest that clinicians’ acceptability of the CFI depended not only on the content of these questions but also on how comfortably they could be introduced and negotiated within the clinical interaction.

### 3.3 Utility

#### 3.3.1 CFI deepened contextual, person-centered understanding of the patient

As discussed earlier, the rapport and relational benefits of administering the CFI enhanced its acceptability. In addition, clinicians across the FTS and PFTS recognized the utility of these same relational benefits in deepening contextual understanding and facilitating person-centered care.

Clinicians across settings described this contextual understanding as strengthening the therapeutic alliance in different ways. Clinicians from the FTS acknowledged that the contextual clarity provided by the CFI facilitated rapport building. Clinicians from the PFTS reported several factors contributing to alliance building. For instance, Mexican clinicians highlighted that authentic information-sharing and feeling heard led to rapport formation, whereas clinicians from Denmark and Sweden attributed this to the collaborative process facilitated by the CFI, which empowered patients. They explained that the patient-provider power imbalance was reduced, as the CFI provided an opportunity for patients to share their insider knowledge regarding culture, religion, and origin, as well as previously undisclosed information. This open-ended format enabled attentive and empathic interaction, and thus rapport.

Swedish clinicians (PFTS) particularly reported that the CFI encouraged clinicians to be curious, exploratory, and collaborative, and to engage in deeper listening, thus eliciting nuances of daily life, stressors, identity, and distress. This process enhanced the utility of the CFI, as it increased clinicians’ grasp of patients’ interpretation of their illness. This was reflected in documentation practices, where clinicians began recording notes more in patients’ own words than through clinician reinterpretation.

However, these benefits were not universal. Clinicians from the Netherlands (FTS) and Denmark (PFTS), voiced counterpoints. The Dutch participants expressed skepticism, perceiving that the CFI’s structured nature could diminish the alliance. Danish clinicians saw this benefit as conditional, since difficult questions on cultural identity risked irritating patients and harming the therapeutic relationship rather than helping it. These findings suggest that the CFI’s contribution to person-centered care depended not only on the information elicited but also on how the interview was conducted, and the contextual background of the clinicians.

#### 3.3.2 The contribution of the CFI to diagnosis and treatment planning remained inconsistent

While the CFI was lauded by clinicians for its potential to enhance person-centered care, they voiced mixed opinions on its potential to aid diagnosis, with three broad perspectives emerging across the included studies.

First, clinicians acknowledged that contextual information aided in clarifying and increasing confidence in diagnosis (India and Pooled, FTS). Clinicians from the PFTS similarly reported that contextually rich information sharpens diagnostic reasoning, as it gives them access to information about the nature, causes, and meaning of distress, care needs, and the nuances of patients’ daily lives, social networks, and cultural backgrounds (Mexico and Sweden, small subset of Dutch clinicians). This information further supported treatment planning by flagging areas for further exploration and identifying coping mechanisms, treatment preferences, support systems, and likely adherence (Sweden).

Second, clinicians also reported persistent uncertainty about translating information elicited from the CFI into a diagnosis. For instance, Dutch clinicians (FTS and PFTS) clearly reported this doubt, questioning the utility of the CFI in diagnosis-making at all. It is worth noting that this uncertainty was consistent across two independent Dutch samples. The Dutch clinicians from the PFTS added nuance to this position, noting that the CFI was more useful for personality disorders but may not hold value for symptom-based conditions such as anxiety, depression, or ADHD. A similar view was reported by Danish clinicians, who appreciated the profound understanding the CFI offered of migrant patients’ experiences but found it difficult to translate this understanding into diagnostic value of any kind.

Third, USA clinicians (FTS) occupied a distinct intermediate position that did not align neatly with either pattern. Although they did not strongly emphasize the CFI’s diagnostic value, they expressed a preference for using targeted supplementary questions when diagnostic uncertainty persisted, reflecting a conditional rather than categorical view of its diagnostic utility. These findings suggest that while clinicians value the information elicited through CFI, there are considerable clinician differences in perceiving the utility of the CFI for diagnostic reasoning and treatment planning.

#### 3.3.3 Clinical utility depended on clinician’s conceptualization of culture

Clinicians across studies questioned what “culture” and “cultural identity” represented as clinical constructs and whether the CFI operationalized these concepts in ways that were meaningful for routine psychiatric practice. This is related to but also conceptually distinct from the discomfort discussed earlier. This dimension seems to represent a more fundamental question about the CFI. The clinicians from the USA (FTS) perceived culture as an anthropological construct rather than clinical and questioned whether information concerning family relationships, social context, idioms of distress, or clinicians’ own backgrounds constituted cultural information. They also observed that the information elicited via these questions was more akin to personal information than cultural. They reasoned that the contextual information may be meaningful for the context of trial than clinical practice, and, in that sense, the CFI was seen to be shaped by anthropology rather than clinical practice. Similarly, a subset of Indian clinicians also viewed culture as not relevant to their routine practice, viewed the CFI as complementary and not a primary diagnostic tool.

Comparable uncertainty was evident in the PFTS, although clinicians more frequently described strategies for negotiating these conceptual challenges during interviews. It was noted that the questions on culture either resulted in no, limited or overly personal information in response. Consequently, clinicians routinely expanded, rephrased, or contextualized these questions to fill in the gap. Most often, this was visible in questions centered around the cultural identity of the patients.

One Mexican clinician reasoned that perhaps personal information such as family roles, religion, gender, education, employment and place of residence, represented what constituted culture in practice. Thus, they needed to expand the definition of culture for their patients using these words. In contrast, Danish clinicians also expanded the definition of culture when patients didn’t understand the question, but expressed concern that doing so might inadvertently introduce clinicians’ own biases. They therefore suggested that the quality of information obtained depended less on the instrument itself than on clinicians’ training and capacity for critical reflection. Finally, clinicians from Sweden reported these questions to be novel, appreciating the personal information elicited even as it extended beyond conventional markers such as ethnicity or migration.

#### 3.3.4 Clinician factors shaped the CFI’s perceived utility

Clinicians’ pre-existing assumptions, cultural competence, professional experience, and personal dispositions consistently influenced how the CFI was administered and the extent to which it was perceived as clinically useful. This theme emerged most clearly, and almost exclusively within the post-field-trial studies.

Many clinicians entered interviews with preconceived expectations regarding the circumstances under which cultural information would be relevant. Cultural issues were often anticipated primarily among patients from rural backgrounds or ethnic and racial minority groups. For example, clinicians in Mexico expected discussions of supernatural beliefs, indigeneity, and ethnicity, whereas Danish clinicians anticipated obtaining “insider information.” Consistent with this perspective, Danish clinicians also questioned the usefulness of the CFI for symptom-based disorders. These preconceptions appeared to influence how clinicians interpreted patients’ responses, the areas they chose to probe further, and the extent to which they integrated cultural information into diagnosis and treatment planning. Consequently, the perceived utility of the CFI appeared to depend not only on the instrument itself but also on clinicians’ existing conceptualizations of when and where culture was considered clinically relevant. Supporting this interpretation, Dutch clinicians with greater cultural competence and more years of professional experience were better able to develop accurate cultural formulations, suggesting that clinician expertise potentially shaped the CFI’s perceived effectiveness.

Clinician disposition also influenced the administration of the interview. Some clinicians reported that the CFI prompted exploration of culturally relevant issues that might otherwise have remained unaddressed and encouraged reflection on their own implicit assumptions about patients’ cultural backgrounds. In contrast, others expressed concern that an emphasis on cultural sensitivity could inhibit clinically important enquiry, with fears of appearing stigmatizing leading them to avoid questions related to culture, family support, guilt, or shame. Collectively, these findings suggest that the CFI’s implementation and perceived utility were shaped as much by clinicians’ prior knowledge, skills, and attitudes as by the instrument itself.

### 3.4 Thematic overlaps

Mapping clinician narratives across the FAU evaluative framework of the CFI reveals that the FAU domains are highly interdependent, with several thematic patterns recurring across multiple evaluative domains. Because the FAU domains were mapped and analyzed separately for FTS and PFTS, this synthesis cannot establish directional or causal relationships between themes - for instance, whether clinicians’ concerns about rigidity in section 3.1 *caused* the discomfort reported in section 3.2, or whether the two simply co-occurred in the same narratives. What the data do allow is identification of recurring patterns that surfaced in more than one domain, suggesting they function as threads running through clinicians’ reported experiences of the CFI rather than being confined to a single evaluative dimension. Several distinct cross-cutting patterns were observed.

One recurrent pattern was that time constraints are tolerated only when clinicians perceive high clinical return on investment. Across studies, clinicians appeared to weigh the additional time and effort required to administer the CFI against its perceived clinical value. Within the feasibility domain, this tension was expressed primarily in relation to consultation time and workload (3.1.1), whereas within the utility domain it centered on whether the information elicited improved diagnostic reasoning or treatment planning (3.3.2). These findings suggest that many clinicians evaluated the utility of the CFI in terms of its contribution to diagnostic clarification and treatment planning, rather than solely its capacity to enhance person-centered understanding.

Rapport recurred as a precondition for feasibility (Indian FTS clinicians questioning whether sufficient rapport must exist before the CFI could be administered), a driver of acceptability (3.2.1), and a mechanism of utility (3.3.1). Another recurring cross-cutting theme is the struggle to reconcile the CFI’s standardized structure with the intuitive nature of clinical practice. Reflecting this structural tension, clinicians across studies used informal adaptations (rephrasing, selective administration, drifting) to preserve both feasibility and clinical utility.

Finally, the synthesized data demonstrate that clinicians’ experiences with the CFI were mediated by clinician-level characteristics and local institutional contexts. Individual factors such as prior professional experience, baseline cultural competence, and personal disposition, consistently shaped their evaluation on FAU (3.1.2, 3.2.1, 3.3.4). Furthermore, the construct of “culture” itself operated across domain boundaries, manifesting as interactional discomfort during cultural-identity inquiries (Acceptability, 3.2.2) and as ambiguity regarding whether the information elicited constituted clinically meaningful cultural understanding (Utility, 3.3.3).

## 4 Discussion

Existing literature on the quantitative data of the Feasibility, Acceptability, and Clinical Utility (FAU) of the Cultural Formulation Interview (CFI) has shown clear trends of clinicians’ acceptability being the highest, followed by clinical utility, with feasibility as the lowest-performing domain (13). The present review takes these quantitative findings further by mapping qualitative clinician narratives from field-trial studies (FTS) and post-field-trial studies (PFTS) onto the same evaluative domains, examining not only what clinicians reported but how and why they experienced the CFI as they did.

Across both bodies of literature, clinicians consistently valued the CFI for strengthening therapeutic alliance, facilitating person-centered understanding, and eliciting contextual information that might otherwise remain unexplored. However, these perceived benefits were tempered by persistent concerns regarding its feasibility in routine practice, discomfort with certain interview questions, uncertainty about the clinical value of the information elicited for diagnosis and treatment planning, and differing conceptualizations of culture among clinicians. We found a recurring and underexamined paradox: clinicians could elicit rich cultural narratives from patients, yet frequently remained uncertain whether such narratives constituted cultural information and what to do with them clinically. The present review suggests that this paradox - rather than logistical feasibility concerns alone - may be a central driver of the persistent implementation gap. In this sense, the present review suggests that culture may be hidden in plain sight - present within routine clinical encounters, elicited through the CFI, but not recognized, named, or utilized as such.

The following section discusses the significant patterns in higher-order themes, namely the comparison of the FTS and PFTS, the conceptual problem of culture, and the utility paradox.

### 4.1 Comparison of field trial and post-field trial studies

Organizing clinician narratives within the FAU framework and comparing them across the FTS and PFTS corpora - which differ in study purpose, clinical context, training conditions, and geographical setting- reveals a striking pattern of thematic persistence alongside meaningful differences in the scope and reflexivity of clinician accounts.

Seven of the ten themes identified in this review were present across both corpora (**Table 2**). Regardless of the setting, study design, version of instrument or training conditions, these seven themes recurred across the data set. The persistence of these themes across such varied contexts is notable. It suggests they are not incidental implementation problems resolvable through minor adjustments to the instrument or its delivery, but rather structurally embedded tensions between the CFI’s person-centered logic and the diagnostic orientation of routine psychiatric practice. Newer implementation efforts, including the CFI-Engagement Aid (24) and the Sociocultural Formulation Interview (20), represent meaningful attempts to address these concerns, yet the themes persisted across studies conducted after many of these developments, further supporting the view that instrument refinement alone is insufficient.

One theme was present in the FTS but did not emerge as a distinct pattern in the PFTS. Patient-related factors- particularly concerns about administering the CFI with patients experiencing psychosis, cognitive impairment, or low educational levels- were prominent in the USA field trial but were largely absent from post-field trial accounts. Whatever the explanation, its disappearance from PFTS accounts is worth noting as a potential indicator of evolving clinician orientation toward the CFI’s applicability.

Two themes emerged distinctly in the PFTS that were not foregrounded in the FTS. The first was systemic and organizational barriers- including lack of institutional commitment, competing priorities, inadequate training infrastructure, and difficulties communicating CFI findings within multidisciplinary teams. The field trial operated under standardized, researcher-supported conditions that insulated clinicians from many of these pressures. When the CFI moved into routine practice without that scaffolding, the systemic dimension became visible as a distinct implementation layer. This pattern is consistent with broader implementation science literature, which notes that challenges invisible under controlled trial conditions frequently become prominent during real-world scale-up (25). The second emerging theme was clinician factors shaping utility- including prior cultural competence, professional experience, personal disposition, and conceptualization of culture- which in the PFTS became a central explanatory variable rather than background variation. In the field trials, clinician variability was observed but not foregrounded analytically; in post-field trial studies it was increasingly named as a mechanism through which the CFI’s effectiveness was mediated.

This shift is significant. It suggests that as the CFI has moved from controlled research conditions into routine clinical practice, the field’s understanding of what drives implementation success has deepened- from instrument characteristics and logistical barriers toward the more complex question of who is administering the CFI and with what conceptual and reflexive resources. Importantly, while the problems identified across both corpora are largely the same, what has changed is the field’s capacity to articulate where those problems originate. This sets up the central argument of the sections that follow: that the persistent implementation gap may be less about the CFI as an instrument and more about the conceptual and pedagogical conditions under which it is used.

### 4.2 ‘Culture’ as a conceptual and contextual problem

An important finding across studies, synthesized in Section 3.4, was the complexity of clinicians’ experiences, especially around the concept of “culture” itself. Clinicians’ own cultural contexts and assumptions appeared to shape how they expected culture to “show up” in interviews, which forms of clinical data they identified as cultural, and even their comfort, hesitation, or biases in approaching such topics. This was further complicated by clinicians’ difficult experiences with the cultural identity questions, which persisted even with the published version of the CFI. These questions often required clinicians to move beyond the script and modify the wording in order to elicit responses they experienced as meaningful or clinically relevant.

Perhaps the most striking finding of this review was that clinicians consistently elicited information that reflected patients’ social worlds, relationships, explanatory models, and lived experiences, yet often struggled to recognize or conceptualize these narratives as “cultural” information. In this sense, culture appeared to be hidden in plain sight. Rather than reflecting an absence of cultural material, clinicians’ accounts suggested uncertainty regarding what constituted culture and how such information should be interpreted within psychiatric assessment.

It was observed that patients would more often than not share information about their family, education, employment, and other interpersonal relationships. These responses continued to perplex clinicians in both field-trial and post-field-trial studies across locations. For instance, clinicians from the USA (FTS) questioned whether patients’ accounts of family, education, employment, and interpersonal relationships constituted “cultural” information, perhaps because these narratives did not align with their expectations of culture as relating primarily to race, ethnicity, religion, or language. This is reflected in statements such as, *‘It’s aimed at getting cultural information … but I’m not hearing themes of race, ethnicity, culture, religion … I don’t know how you guys are defining culture…’* (13, p.11).

This same confusion played out differently across countries. Mexican clinicians (PFTS) recognized this information as important within their context, but did not necessarily identify it as cultural information. Danish clinicians worried that such questions could lead patients to feel “othered.” In contrast, Swedish clinicians readily recognized this information as relevant to all patients and regarded them as an important means of understanding patients’ cultural contexts. This may also invite reflections on whether clinician perceptions (or misperceptions) of culture stem from their own professional, institutional, and sociocultural contexts.

Context indeed appeared to shape what clinicians emphasized in the CFI. Dutch field trial publications foregrounded findings related to immigrant patients as a distinct analytic focus, whereas qualitative findings from India more prominently emphasized family presence during assessment, hierarchy within clinical interactions, and constraints related to time and resources. Notably, with the exception of the Indian site, clinician narratives across studies rarely referenced the presence of relatives or family members during CFI administration- a finding worth reflecting upon, given that the CFI includes a dedicated Informant Version designed precisely to incorporate the perspectives of those accompanying patients. That this version received so little attention in clinician accounts outside India may itself reflect contextually shaped assumptions about who the relevant subject of cultural assessment is- the individual patient alone, or the patient within their relational and familial world.

Interestingly, as noted elsewhere, in the Indian context (17), most patients did not consider cultural background differences of clinician-patient relationships to be relevant. This finding itself reflects a form of cultural specificity: in a context where clinicians and patients often share a broad sociocultural framework, even across caste, regional, or linguistic variation, questions regarding cultural background may feel less alien or threatening. At the same time, the Indian context introduced its own challenges, notably around hierarchy and reflexivity. Paralikar et al. (17) documented two instances where clinicians answered “not applicable” to questions about their own cultural background, a finding that speaks directly to the difficulty of reflexivity within a professional culture that constructs the clinician as a neutral expert rather than a culturally embedded subject.

Rather than representing random variability, these differences reflect how clinicians themselves are culturally and institutionally situated. Their professional training, institutional practices, and sociocultural assumptions influenced how the CFI was understood and enacted. Similar observations have been reported in broader cultural psychiatry literature, where cultural consultation services across Montreal, London, and Paris differed substantially in their categorization of diversity and organization of intake procedures, reflecting the influence of local institutional histories and professional cultures on psychiatric practice (26).

In many clinical and biomedical contexts, culture is often understood as a static identity, as a checklist of sociodemographic variables such as religion, ethnicity, race, and caste (5, 14). This narrower conceptualization may partly explain why clinicians across studies struggled to recognize family relationships, employment, education, and everyday illness experiences as forms of cultural information, despite these being central to the CFI’s broader understanding of culture. However, findings from the post-field-trial studies also suggested some expansion in clinicians’ conceptualizations of culture. Even where narrower definitions persisted, clinicians often appeared more reflective about these conceptual limitations.

Interestingly, relatively little attention has been paid within the implementation literature to clinicians’ own conceptualizations of culture as a potential influence on CFI implementation. Few studies account for the local and contextual variations of how culture is defined, experienced, and understood by clinicians, with many studies taking for granted that clinicians across the globe and professions see culture the same way CFI does. Differences in clinician experiences have more commonly been interpreted in relation to feasibility, training, or organizational constraints than to variation in how culture itself is understood. Yet the present review suggests that these conceptualizations may shape how clinicians recognize, elicit, and interpret sociocultural information during assessment.

This tension has a longer history, as suggested by Aggarwal (2024) who traces how culture in successive DSM editions was used primarily to refer to the beliefs and behaviors of minority groups, consistent with demographic constructions of race and ethnicity, before anthropological critics argued that this conception stereotyped populations without accounting for individual variation and treated culture as a static variable rather than a dynamic process of meaning-making in which all individuals - including clinicians- are engaged (15). That clinicians in the present review continued to reflect this narrower conception despite the CFI’s broader anthropological definition suggests that the conceptual revision embedded in the instrument has not been reliably transmitted through training.

Importantly, the challenge identified in this review was not that clinicians failed to elicit cultural information, but that they frequently struggled to recognize ordinary clinical narratives as expressions of culture. Thus, culture appears to remain hidden in plain sight, not only within routine clinical encounters but also within efforts to understand how the CFI is implemented across settings.

### 4.3 From cultural information to clinical utility

A prominent theme across the reviewed studies was clinicians’ difficulty in translating cultural information into clinical formulation and treatment planning. When clinical value was recognized, it most often related to a supporting role in diagnosis rather than to broader formulation or intervention. For instance, clinicians from the Netherlands expressed the view that cultural information was less relevant when symptoms were considered primary- as in ADHD or anxiety- while clinicians from Mexico valued the CFI primarily for the information it yielded about patients’ social support systems, which held particular relevance within their clinical and cultural context. The difficulty, therefore, did not always stem from resistance to contextual information itself but from uncertainty about its clinical function- what it was for, and how it connected to the clinical decisions that followed. This uncertainty may partly explain the lower perceived utility reflected in the qualitative narratives and suggests that the central implementation challenge may be less about reduced feasibility than about an unresolved tension between the effort of administration and the perceived clinical return.

Aggarwal (15) observed a related paradox: despite the CFI’s introduction being intended to support cultural case formulation, fewer cultural formulation case reports have been published after its publication than before- suggesting that providing a structured interview does not automatically translate into formulation practice. He also notes that neither DSM-5 nor DSM-5-TR specifies how to produce a cultural formulation from CFI data- a structural absence that may partly account for clinicians’ uncertainty about the clinical function of the information they elicit. The present review’s findings offer a partial explanation: clinicians who remain uncertain about what constitutes cultural information, and how to interpret it within a biomedical clinical framework, are unlikely to produce cultural formulations regardless of the instrument available to them. Indeed, the instrument provides the questions; it does not provide the reasoning framework for what to do with the answers.

Attempts have been made to address these issues. Weiss et al., for instance, in their sociocultural framework/formulation interview (SCFI), introduced a summary domain to support clinicians in formulation by guiding clinicians to summarize the significant sociocultural features along with their implications for rapport, diagnosis, and case management (20). These changes led to an increase in overall ratings of the clinicians for clinical relevance and implications on treatment. However, the SCFI’s broader promise remains to be tested across independent clinical settings and diverse populations.

Importantly, our review of qualitative findings suggests that the clinical utility of CFI relied heavily on clinicians’ ability to use the tool effectively. With this background, we agree with the suggestions that consider CFI not only as an intervention, but also as a pedagogical tool (3). Aggarwal (2024) similarly concluded that examining how clinicians use CFI information in clinical tasks - and what training and supervision are needed to support this- represents a priority for future research, a direction the present review’s findings both affirm and extend (15).

Cultural assessment differs from conventional diagnostic inquiry insofar as it positions patients as experts of their own cultural worlds, requiring clinicians to adopt a stance of epistemic humility and inquiry. Therefore, the CFI holds pedagogical value that extends beyond its immediate clinical outputs - and training that cultivates these capacities holds potential clinical value irrespective of the patient’s diagnosis or background.

## 5 Recommendations: an interpretive synthesis

As described in Methods, section 2.4, we performed a synthesis of recommendations proposed across the literature, and an interpretive analysis of broader conceptual tensions emerging from the dataset. The following section presents the outputs of both activities, organized around their implications for clinical practice, training, and future research. Section 5.1 synthesizes recommendations proposed across the included literature, organized by degree of change implied. Sections 5.2 and 5.3 present our own propositions, grounded in the interpretive analysis described in section 2.4, where the present findings extend what has previously been proposed or point toward directions not yet adequately addressed.

Our review findings prominently underscore the clinicians’ interest in the CFI, but the implementation is influenced by their ambivalent experience with the tool. On one hand, clinicians are appreciative of the person-centered *process* facilitated by the tool, but remain uncertain about the clinical utility of the *content* elicited, and sometimes find it difficult to even elicit the content, especially for the questions on cultural identity, thus explaining the origin of conundrums partly. However, evidence suggests that increased clinical experience and familiarity with the tool significantly improve clinician evaluative ratings over time (1).

Two broad recommendations (5.2 and 5.3) emerge from the literature and from our own findings. The first concerns clinicians’ own conceptualization of culture and the second, the development of the clinical skills needed to bridge the gap between sociocultural information and the biomedical frameworks within which most clinicians practice. Both directions are explored in the sections that follow.

### 5.1 Synthesis of recommendations in literature

Several suggestions and efforts have been made to address the implementation barriers of the CFI to help improve the clinical uptake. It is interesting to note the multiple approaches recommended for similar problems, thus demonstrating several possibilities and potentials to further develop the CFI. We noted that the suggestions and recommendations can be categorized into the following categories and are summarized in **Table 3**:

#### 5.1.1 Minimal refinements

Referred to the recommendations that largely keep to the structure and the content of the existing CFI model. Most of the recommendations in this domain were made with respect to modifications to wording/questions of the CFI, such as the rephrasing or reframing of the cultural identity and background questions (5, 7, 16, 27) and expansion of prompts to include broader cultural dimensions such as family roles, education, gender in the CFI process (2). Some also recommended linguistic and contextual adaption of CFI (13, 21) or an abbreviated version (9, 17). Many of the initial recommendations belonged to this category.

#### 5.1.2 Moderate adaptations

Are indicative of suggestions referring to additions and modifications to the existing CFI model. Most often, modifications were suggested to the implementation/training/workflow involving the CFI (2, 5, 13, 22, 23). Recommendations related to training, supervision, and flexible administration emerged most consistently across studies and countries, suggesting a broad consensus that implementation challenges are not solely attributable to the CFI instrument itself but also to clinician preparation and contextual integration (2, 5, 9, 24). Interestingly, most recommendations appeared to cluster within the moderate adaptation category.

#### 5.1.3 Transformative/paradigm-level/major recommendations

These substantially reconceptualize the existing CFI model or the clinical practices in mental health. These included suggestions to modify the epistemology or structure of psychiatric assessment itself. More recent literature increasingly proposes broader systemic and epistemological reforms, including person-centered assessment models, structural competency approaches, and therapeutic assessment frameworks. While a few experts discuss situating CFI within the broader psychiatric assessment (13, 27), more radical recommendations have been made by more recent authors who suggest decentering the CFI or shifting the general models and styles of psychiatric assessment.

We also noted that beyond recommendations, efforts have been made to improve the translation of sociocultural findings into clinical practice through implementation aids, formulation frameworks, and consultation-oriented models, including the Cultural Formulation Interview-Engagement Aid (24), the McGill Cultural Consultation Service (CCS) model (28, 29), and the domain-based Sociocultural Formulation Interview (SCFI) (20) approaches, which support clinicians in organizing, interpreting, and integrating sociocultural information into formulation and treatment planning.

Several patterns are worth noting across these recommendations. First, recommendations regarding the cultural identity questions (Q8–10) represent the single most consistent target for minimal refinement, with researchers and clinicians across every included context identifying these questions as requiring rewording, expanded prompts, or clearer framing - suggesting a cross-cultural consensus that is unusual given the otherwise highly context-dependent nature of implementation challenges. Second, the moderate adaptations category is not only the most populated but the most actionable, containing the clearest near-term roadmap for implementation improvement: standardized training combining didactic and experiential components, ongoing supervision, fidelity assessment, explicit guidance for translating findings into formulation, and institutional embedding. Third, transformative recommendations appear predominantly in the more recent literature, suggesting that as the field has accumulated implementation experience, its ambitions for what needs to change have grown correspondingly broader. Finally, one productive tension within the synthesis is worth acknowledging: recommendations to reconsider universal routine administration of the CFI sit alongside recommendations to actively dismantle the perception that the CFI is relevant only for minority or migrant patients. These are not straightforwardly compatible positions, and resolving this tension - between context-sensitive deployment and universal cultural relevance - represents an important conceptual task for the field going forward.

Our reading of the present findings aligns most closely with the transformative end of this spectrum. The thematic persistence documented in this review - across contexts, instrument versions, and levels of implementation investment - suggests that the implementation gap cannot be fully addressed through instrument refinement or training additions alone. The deeper challenge, as the preceding sections have argued, concerns clinicians’ conceptualizations of culture and their capacity to recognize, interpret, and act upon sociocultural information within routine clinical practice. Addressing this requires not only better tools but a different orientation and understanding toward what psychiatric assessment is for.

### 5.2 Conceptual clarity on ‘culture’

A central priority emerging from the present findings is greater conceptual clarity regarding culture within CFI training. The present review consistently identified clinicians’ narrow or static conceptualizations of culture - organized around ethnicity, race, and religion rather than dynamic, relational, and intrapersonal experience - as an underexplored mediator of implementation difficulties.

Clinicians across studies entered interviews with preconceptions about what kind of information would emerge and for whom- cultural material was anticipated primarily among patients from minority or migrant backgrounds. Their prior exposure to multicultural clinical settings appeared to shape how they navigated this uncertainty: in Sweden, where clinicians had substantial multicultural experience and a broader professional orientation toward context, appreciation for this information increased; in Denmark, where sociopolitical anxieties around naming cultural difference were prominent, the same questions generated discomfort and relational distance rather than deeper understanding.

This is not merely a knowledge gap but a conceptual one: clinicians were not simply unaware of a broader definition of culture but were operating within professional and institutional frameworks that actively organized their clinical attention around symptom categories rather than contextual ones. This pattern can be observed in literature as well, where culture is narrowly defined and conceptualized, unintentionally contributing to essentialist assumptions and practices and potential discrimination and othering within clinical encounters. The DSM-5 (30) DSM-5 itself partially reflects this tension by defining culture as “systems of knowledge, concepts, values, norms, and practices that are learned and transmitted across generations. Culture includes language, religion and spirituality, family structures, life-cycle stages, ceremonial rituals, customs, and ways of understanding health and illness, as well as moral, political, economic, and legal systems” (p. 749). While the DSM-5 additionally acknowledges culture as dynamic, heterogeneous, and shaped by multiple social contexts, its emphasis on socially shared and transmitted group characteristics may inadvertently encourage categorical or group-based understandings among clinicians whose training has not otherwise foregrounded anthropological or sociological frameworks.

Aggarwal (15) notes that DSM-5-TR extended the DSM-5 definition precisely to address this limitation, adding language recognizing that cultural meaning-making derives from developmental and everyday social experiences in specific contexts, including healthcare. Drawing on the findings of this review and broader cultural psychiatry scholarship, we contemplated another operationalization of the concept of culture. The proposed conceptualization in the present review builds on the DSM trajectory, extending it further toward an explicitly intrapersonal and relational understanding that foregrounds individual agency in the internalization of cultural influences- and that applies equally to clinicians and patients.

We tentatively propose the following conceptualization, grounded in the patterns identified across this review and existing cultural psychiatry scholarship.

“Culture comprises the evolving and dynamic ideas, values, norms, and practices within a person’s context and environment that are identified with, negotiated, and internalized over the course of life, which influence their experiences, meaning making, choices, decisions, and interpersonal-relationships.

These cultural processes emerge through the interaction of factors including, but not limited to, gender, language, place, race, ethnicity, religion, caste, migration, social status, financial status, social groups, political affiliation, work, role within family, stigma, victimization, rituals, and practices.”

This conceptualization broadens the relevance of the CFI beyond its frequent association with ethnic minority or migrant populations, recognizing that all individuals are shaped by sociocultural influences embedded within everyday lived experience. It supports an anti-essentialist approach by emphasizing individual agency in the internalization of cultural influences, encouraging contextual inquiry rather than group-based assumptions. Importantly, because this conceptualization applies equally to clinicians, it foregrounds reflexivity and challenges assumptions of cultural neutrality within psychiatric practice.

A clear conception of culture may help clinicians more effectively identify and utilize cultural information in formulation. A patient expressing guilt about being unable to financially support their family may not simply be describing an individual stressor, but also reflecting internalized values related to gender roles, familial obligation, or socioeconomic pressure. Recognizing such influences may clarify explanatory models of distress, treatment preferences, adherence difficulties, and sources of resilience - making cultural information clinically actionable rather than supplementary and allowing formulation to move beyond symptom description toward a more person-centered and contextual understanding of the patient.

However, even if the anti-essentialist idea of culture has been discussed in research, it is unclear how aware clinicians are of these ideas. Most clinicians have access to the DSM-5–16 question CFI document, which does not mention the definition. Our review of the training clinicians receive (**Table 1**) seems to indicate that the discussion of culture may not be in-depth for many clinicians trained in the CFI. Training that does not explicitly address what culture means within the CFI framework- and how it differs from common clinical and lay understandings - is unlikely to equip clinicians to recognize the sociocultural information already present in their routine encounters. These divergent responses to a shared conceptual challenge point to a strong and context-sensitive need for explicit conceptual clarity in CFI training - one that addresses not only what culture means within the CFI framework but what clinicians already believe it to mean, and why. Training that does not start from that diagnostic question is unlikely to shift the conceptual assumptions that our findings suggest are driving a significant portion of the implementation gap.

### 5.3 Towards socio-culturally informed clinicians

A second priority concerns the operationalization of the clinical competencies involved in sociocultural assessment. Clinicians’ prior experience, conceptualization of culture, reflexive orientation, and professional background influenced how they used the CFI, what they considered culturally salient, and how they translated the information into clinical practice (sections 3.3.3, 3.3.4, 3.2.2, 3.1.2). Thus, the value derived from the CFI potentially depends less on the instrument itself than on the competence of the clinician administering it.

As reflected in the moderate and transformative recommendations synthesized in **Table 3**, the field has increasingly recognized that implementation challenges are not solely attributable to the instrument itself. Existing frameworks have made important contributions here. Cultural competence frameworks have described the knowledge, attitudes, and skills required for working across cultural differences (31, 32). Cultural humility, as proposed by Tervalon and Murray-García, extended this by emphasizing ongoing self-reflection and the redressing of power imbalances within clinical encounters (32). Structural competency, as articulated by Metzl and Hansen, added attention to systemic and institutional factors shaping patient distress (33). More recently, the Sociocultural Formulation Interview framework proposed by Weiss et al. has attempted to operationalize these principles within the clinical encounter itself (20). These frameworks offer a meaningful foundation and inform several of the training and supervision recommendations captured in **Table 3**.

However, what remains underdeveloped, especially for the CFI, is explicit guidance on how these competencies translate into the specific clinical behaviors involved in recognizing, eliciting, interpreting, and utilizing sociocultural information during psychiatric assessment - the granular clinical skills that the CFI demands but that existing training models do not yet systematically address. The results from our review also imply that implementation challenges may persist because sociocultural assessment cannot rely solely on procedural administration of interviews or checklists.

Perceived procedural rigidity (feasibility) and low diagnostic yield (utility) likely stem from the same underlying training gap - explaining why both feasibility and evaluative ratings improve as practice increases (1). Future implementation research could test this directly using the CFI-Fidelity Instrument (24) alongside parallel feasibility and utility ratings to determine whether training is the common mechanism underlying both outcomes.

Considering this background, we therefore propose a shift toward developing training frameworks that support clinicians in becoming “socio-culturally informed” in routine clinical practice through the internalization of sociocultural frameworks, the development of skills for identifying and utilizing sociocultural data, and the incorporation of supervision practices that support these competencies.

Such a training framework may build upon the reflexivity and curiosity of the clinicians and help them recognize and map cultural information to relevant domains of the CFI. Rather than relying on rigid, checklist-style administration, this approach emphasizes internalizing sociocultural frameworks so they become as organic to clinical reasoning as standard biomedical and psychological models. This nature of practice is organic to clinicians as they routinely practice clinical assessments of diagnosis and treatments based on internalized biomedical and psychological frameworks, rather than structured assessments. Clinicians also identified the need to translate cultural information into clinically actionable decisions, alongside practical difficulties in documentation and communication of CFI findings within multidisciplinary teams. Hence, training needs to explicitly focus on bridging this cultural-clinical gap by operationalizing the skills required for this, encouraging case study publications, more formulations, peer-review and supervision.

## 6 Limitations

The present review was not a formal systematic review and, therefore, may not have captured all published studies examining clinician perspectives on the Cultural Formulation Interview (CFI). The included studies varied substantially in methodology, sample characteristics, clinical settings, clinician backgrounds, and outcome measures, limiting direct comparability across studies. Several studies provided only brief qualitative excerpts or author summaries rather than extensive raw qualitative data, limiting the depth of thematic interpretation and possibly resulting in a fragmented analytic dataset. Because we do not have access to the entire dataset, our paper also inherits the publication bias of all the studies we included because the selected quotes may represent selected findings. Moreover, secondary qualitative analysis risks decontextualizing findings from their original study settings, which may limit the interpretive depth achievable from published excerpts alone.

The review focused specifically on clinician perspectives and implementation experiences; therefore, patient, caregiver, interpreter, and organizational perspectives were not analyzed. The number of post-field-trial implementation studies remains relatively limited, particularly from low- and middle-income countries and non-Western psychiatric systems, restricting broader cross-cultural generalizability. Similarly, the review primarily synthesized English-language publications and may therefore underrepresent implementation experiences and conceptual debates emerging from non-English literature. In fact, the adaptation and utilization of the CFI and other cultural assessments in various local languages are likely to hold the essence of the cultural formulations specific to the locales and the clinical populations. Finally, although the review discusses broader epistemological critiques of psychiatric assessment, the paper does not aim to reject biomedical approaches to diagnosis, but rather to examine how contextual and cultural dimensions may be more meaningfully integrated into existing clinical practice.
